# Avoiding vascular complications in insular glioma surgery – A microsurgical anatomy study and critical reflections regarding intraoperative findings

**DOI:** 10.3389/fsurg.2022.906466

**Published:** 2022-08-05

**Authors:** Gustavo Rassier Isolan, Viviane Buffon, Igor Maldonado, Jander Moreira Monteiro, Kaan Yağmurlu, Carmen Austrália Paredes Marcondes Ribas, Rafael Roesler, Osvaldo Malafaia

**Affiliations:** ^1^ The Center for Advanced Neurology and Neurosurgery (CEANNE), Porto Alegre, RS, Brazil; ^2^ Mackenzie Evangelical Faculty of Paraná, Curitiba, PR, Brazil; ^3^ U1253, iBrain (I.L.M.), Université de Tours, Institut National de la Santé et de la Recherche Médicale, Tours, France; ^4^ Groupe Hospitalier Universitaire Paris, Paris, France; ^5^ Department of Neurosurgery, University of Virginia, Charlottesville, VA, United States; ^6^ Department of Pharmacology, Institute for Basic Health Sciences, Federal University of Rio Grande do Sul, Porto Alegre, Brazil

**Keywords:** Insular gliomas, microanatomy, middle cerebral artery (MCA), skullbase, vascular anatomy

## Abstract

**Introduction:**

Vascular lesions in insular glioma surgery can severely impact patients&apos; quality of life. This study aims to present the results of our dissections and authors’ reflections on the insular vascular anatomy.

**Matherials and Methods:**

The insular vascularization was examined using ×3 to ×40 magnification in 20 cadaveric cerebral hemispheres in which the arteries and veins had been perfused with colored silicone.

**Results:**

In insular gliomas, this individualization of the anatomical structures is rarely possible, as the gyri are swollen by the tumor and lose their individuality. In the transsylvian approaches, the anatomical parameters for delimiting the insula in tumors are best provided by the superior and inferior circular sulci. The branches of the MCA are easily identified in the transcortical approach, but only at the end of the surgery after the tumor is resected.). One of the factors under-discussed in the literature is the involvement of the lenticulostriate arteries by the medial part of the tumor. In our experience of 52 patients (article submitted to publishing), LSTa were founded to be involved by the tumor in 13 cases. In 39 patients, there was no involvement of the LSTa, which allowed a more aggressive resection. Early preoperative identification of the anterior perforated substance on the MRI and its proximity to the tumor may help determine the route of the LSTa over the medial tumor boundaries.

**Discussion:**

Our reflections introduced our imaging and anatomical concept regarding LSTa in insular glioma surgery. Accurate identification of origin, route, and distribution of the LSTa is pivotal to surgical success, especially in the lateral group. The anatomical knowledge of their path directly impacts the extent of tumor resection and functional preservation.

**Conclusion:**

Knowledge of microsurgical anatomy, brain mapping, and surgical experience counts a lot in this type of surgery, creating a reasonable procedure flowchart to be taken intraoperatively.

## Introduction

The insular lobe is known as the “hidden lobe” because it is covered by the temporal and frontal lobes on the lateral surface of the brain. Insular gliomas were considered inoperable until Professor Yasargil's article, in 1992 (1). Over the years and based on the advancement of in-depth knowledge of insular anatomy, development of microsurgery and, more recently, extensive expansion of awake cortical and subcortical mapping in selected cases, the results of insular glioma surgery are highly acceptable, with low morbidity and significant impact on patient survival (2–28).

However, there are still three major concerns about lesions that can impact the quality of life of patients in the postoperative period of insular glioma surgery. The first is the damage to the internal capsule and corona radiata by direct manipulation, which can cause motor and sensory deficits in the contralateral hemibody. The second is the lesion of bundles and fascicles of white fibers surrounding the tumor, such as the inferior fronto-occipital fascicle (IFOF) and superior longitudinal fascicle (SLF). And finally, there is a risk of arterial and venous vascular lesions.

The objective of this study is to present the results of our dissections on the insular vascular anatomy and, based on the case series of one of the authors (GRI), postulate three basic precautions that neurosurgeons should take in surgery in relation to vascularization of the insula.

## Materials and methods

The neural features of the insula vascularization were examined using ×3 to ×40 magnification in 20 cadaveric cerebral hemispheres in which the arteries and veins had been perfused with colored silicone. The course and branches of the internal carotid artery (ICA) and middle cerebral artery (MCA) were studied. The branches arising from the ICA and MCA also were studied. The venous drainage of the sylvian fissure was examined. Illustrative surgical cases were selected from the experience of one of the authors (GRI) in the treatment of insular gliomas, with 55 surgeries for insular gliomas performed between 2007 and 2019.

It is not the purpose of this study to present the results of the case series in detail, as these have already been submitted for publication. Likewise, anatomy is described in terms to support the authors' reflections. Although at the beginning of our series the cases were operated through the transsylvian route, we have recently operated on most of our patients with a transcortical approach and subpial resection with intraoperative cortical and subcortical mapping. This change in surgical technique, however, does not change in any way the anatomical knowledge necessary in the resection of these tumors.

## Results

### Insula

**Figure 1:**
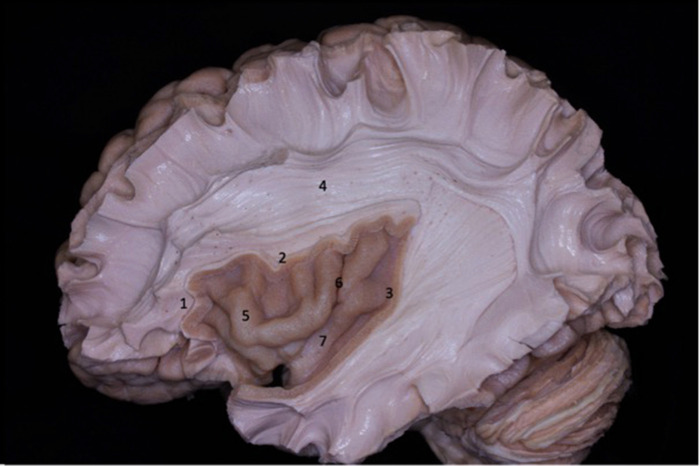
Lateral view of the left cerebral hemisphere after dissection of the cerebral cortex and its u-fibers. The superior longitudinal fasciculus is observed around the insula. The lobe of the insula is pyramid-shaped with its apex oriented anteriorly and inferiorly. 1 - anterior circular sulcus; 2 - superior circular sulcus; 3 - posterior circular sulcus; 4 - superior longitudinal fasciculus; 5 - insular short gyri; 6 - insular central sulcus; 7 - insular long gyri.

**Figure 2:**
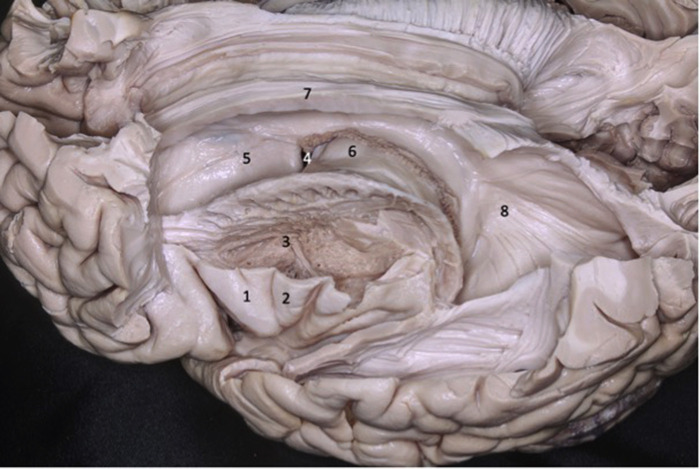
Lateral view of the left cerebral hemisphere. Part of the frontal and temporal lobes were removed to expose the insula, basal ganglia, and ventricular system. The second short gyrus of the insula (2) aligned with the foramen of Monro (4). The foramen of Monro is located medially to the knee of the internal capsule. 1 - first short gyrus of the insula; 2 - second short gyrus of the insula; 3 – basal ganglia; 5 - caudate nucleus head; 6 - thalamus; 7 - corpus callosum; 8 - lateral ventricle atrium.

**Figure 3:**
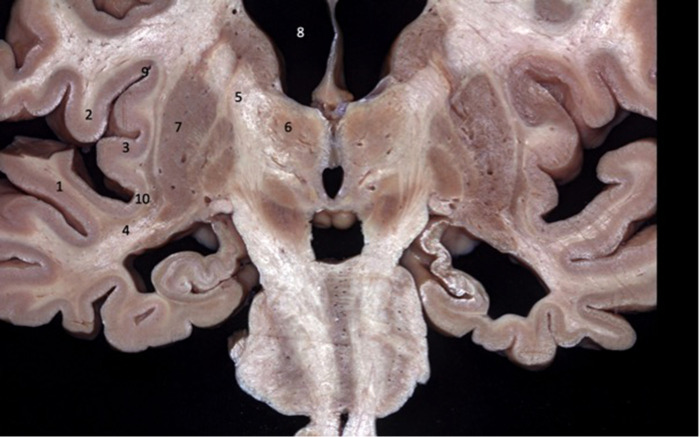
Coronal brain section showing the relations between the insula and the gyri of the sylvian fissure. The superior temporal gyrus (1) covers the lower half of the lobe of the insula while the inferior frontal gyrus (2) covers the upper half. 1 - superior temporal gyrus; 2 - inferior frontal gyrus; 3 - insular cortex; 4 - temporal stem; 5 - internal capsule; 6 - thalamus; 7 - lentiform nucleus; 8 - frontal horn; 9 - superior circular sulcus; 10 - inferior circular sulcus.

The insula has a triangular shape, the vertex of which is called the limen insula and constitutes the lateral limit of the sylvian fissure. The adult human insula is completely hidden within the sylvian fissure on the lateral surface of the brain and only becomes visible after dissection and opening of the fissure. The anterior removal of the fronto-orbital, frontoparietal and temporal opercula reveals the entire insula, with its apex anteriorly and inferiorly oriented. The insula is limited by the anterior, superior and posterior circular sulci and has 4–5 short anterior gyri and 2–3 long posterior gyri, divided by the central sulcus of the insula (Figure 1). The second short anterior gyrus of the insula is located at the level of the intraventricular foramen (of Monro) and corresponds in depth to the genu of the internal capsule, an important site of confluence of the cortico-spinal tract (Figures 2, 3).

### Lateral sulcus or sylvian fissure

The sylvian fissure is a deep, prominent groove that runs through the inferior and lateral surfaces of the brain and extends from the anterior perforated substance to the supramarginal gyrus. It separates the frontal and parietal from the temporal lobe, with the insula as its floor. At its terminal part, the lateral sulcus divides into three branches: ascending, anterior, and posterior. The posterior branch is the longest of the three and runs backwards and upwards, ending in the supramarginal gyrus. The anterior and ascending branches are shorter and divide the inferior frontal gyrus into three parts (from anterior to posterior): orbital, triangular, and opercular. Between the orbital and triangular parts is the anterior branch; and between the triangular part and the opercular, the ascending branch.

The lateral sulcus may also have a fourth branch, called the fronto-orbital, which, when present, crosses the basal surface of the frontal lobe, below the anterior branch and the orbital portion (pars orbitalis).

**Figure 4:**
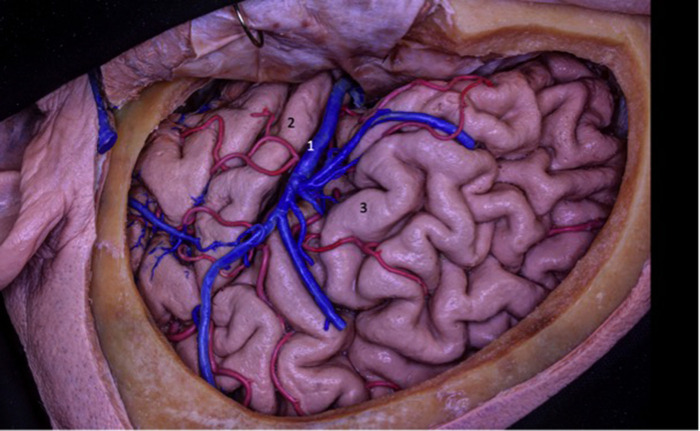
The insula is located on the floor of the sylvian fissure. To access the insula, the fissure must be dissected along its entire length. The insula can also be accessed through corticectomy in the inferior frontal gyrus or superior temporal gyrus, commonly after cortical and subcortical mapping with the patient awake. 1 - superficial sylvian vein; 2 - superior temporal gyrus; - inferior frontal gyrus.

**Figure 5:**
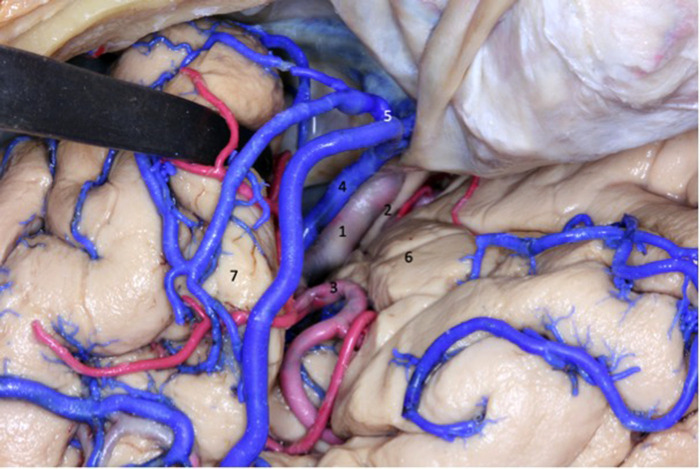
The deep portion of the sylvian fissure is divided into a sphenoid compartment, close to the limen insula, before the sphenoid crest, and another compartment called the insular operculum, formed by two narrow slits: opercular and insular. 1 - internal carotid artery; 2 - optic nerve; 3 - middle cerebral artery; 4 - superficial sylvian complex vein; 5 - superficial sylvian vein draining into the sphenoparietal venous sinus; 6 - inferior frontal gyrus; 7 - superior temporal gyrus.

The sylvian fissure has two portions, one superficial, seen on the surface of the brain, and another deep, hidden below the basal surface, called the sylvian cistern. The superficial portion has a trunk and three branches: anterior horizontal, anterior ascending and posterior, described above. The deep portion, on the other hand, is divided into a sphenoid compartment, close to the limen insula, before the sphenoid crest, and another compartment called the insular operculum, formed by two narrow slits: opercular and insular. The opercular is situated between the lips of the frontoparietal and temporal operculum (Figures 4, 5).

### Insular vascularization

#### Arterial relations

**Figure 6:**
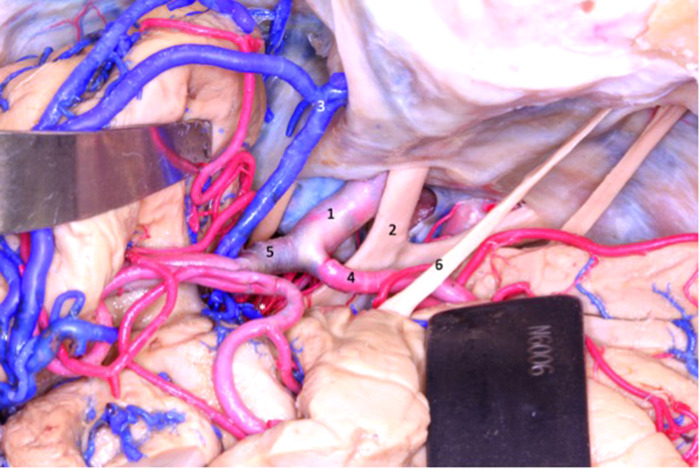
At the beginning of the transsylvian dissection to access the lobe of the insula, the internal carotid (1), anterior cerebral (4) and middle cerebral (5) arteries are exposed. Superficial sylvian complex vein draining into the sphenoparietal sinus (3). 1 - internal carotid artery; 2 - optic nerve; 3 - sphenoparietal venous sinus; 4 - anterior cerebral artery; 5 - middle cerebral artery; 6 - olfactory tract.

**Figure 7:**
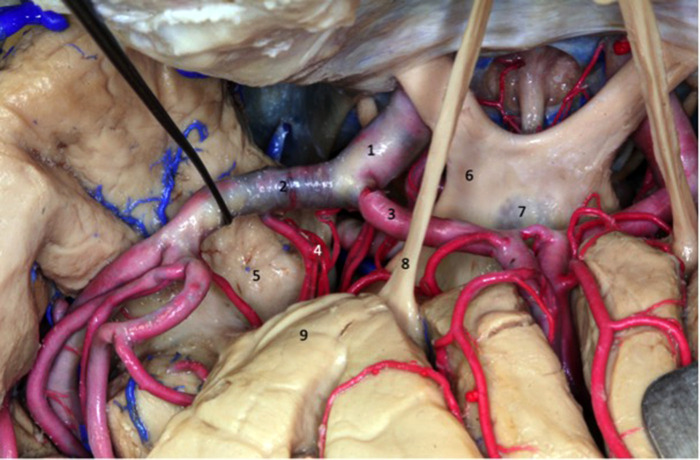
Superior view of the dissected sylvian fissure that shows the arteries that supply the insula. The dissector is laterally displacing the MCA to expose the lenticulostriate arteries (4). 1 - internal carotid artery; 2 - middle cerebral artery; 3 - anterior cerebral artery;4 - lenticulostriate arteries; 5 - uncus; 6 - optic chiasm; 7 - lamina terminalis; 8 - olfactory tract; 9 - frontal lobe.

The middle cerebral artery is the most complex of the three cerebral arteries. It is twice as large as the anterior cerebral artery (ACA). Its origin is located at the beginning of the lateral sulcus, laterally to the optic chiasma (Figure 6). The MCA passes under the anterior perforated substance, into which its small branches called lenticulostriate arteries will penetrate (Figure 7). It then divides into the lateral sulcus and turns postero-superiorly, reaching the surface of the insula. The MCA is responsible for supplying the insula through its branches.

The MCA is divided into four segments, M1 through M4. The M1 segment, also called the sphenoid, has its origin in the bifurcation of the internal carotid artery and the middle cerebral artery and extends to the main bifurcation of the middle cerebral artery, located in the region adjacent to the limen insulae. The M2 segment, also known as the insular segment, extends from the main bifurcation of the middle cerebral artery to the periinsular sulcus. The M3, or opercular segment, begins at the periinsular sulcus and runs through the operculum, ending at the lateral surface of the lateral sulcus. Finally, the M4, or parasylvian segment, corresponds to the branches that supply cerebral convexity.

### M1 segment

**Figure 8:**
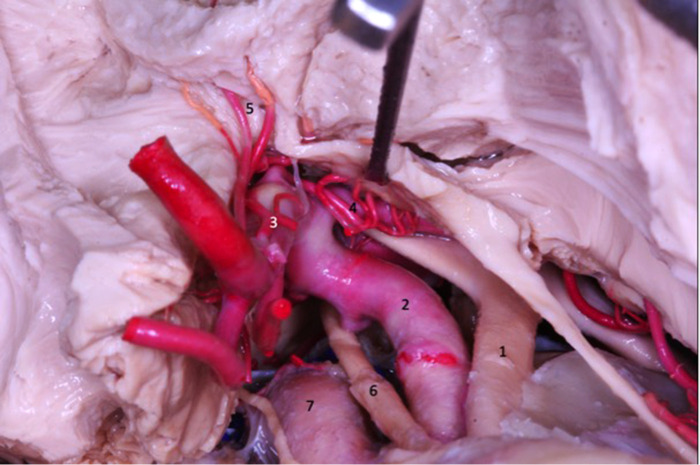
The lenticulostriate arteries (4 and 5) penetrate the anterior perforated substance and ascend to the basal nuclei and internal capsule. 1 - Optic nerve; 2 - internal carotid artery; 3 - anterior cerebral artery; 4 - medial lenticulostriate artery; 5 - medial lenticulostriate artery; 6 - oculomotor nerve; 7 - intracavernous internal carotid artery.

The internal carotid artery forks into the middle cerebral artery and anterior cerebral artery at the level of the central portion of the anterior perforated substance (Figure 8). At the origin of the middle cerebral artery, the M1 segment begins. It traverses the depth of lateral sulcus in an anterosuperior, superior or posterosuperior form, around the limen insulae where it curves. It is here that the end point of M1 is marked by the main bifurcation of the middle cerebral artery into superior and inferior branches. This bifurcation is located mostly in the knee, but it can be proximal or distally to it. In some cases, a trifurcation occurs through the origin of an intermediate trunk which can arise from both the upper and lower trunk. Quadfurcation or pseudotetrafurcation can also occur when both trunks bifurcate, though there are few cases of this in the literature. A “false bifurcation” may also appear, formed by the temporal branch of the M1 segment or by the frontal branch.

The branches of the M1 segment are classified according to the brain areas they supply. Therefore, they can be divided into cortical arteries or lateral lenticulostriate arteries (LLAs).

Regarding the cortical arteries, the M1 segment is divided into pre-bifurcation and post-bifurcation (Pre-bifurcation consists of a single trunk that originates at the beginning of the middle cerebral artery and extends to the M1 segment bifurcation (27). Cortical arteries that arise from pre-bifurcation are called early branches, which correspond to the frontal and temporal branches of the M1 segment. Temporal Were identified in almost all our dissections and frontal branches in 11 hemispheres.

**Figure 9:**
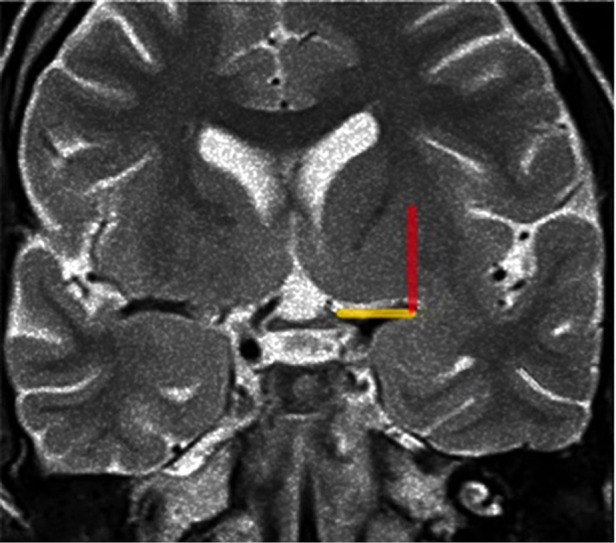
Coronal T2-weighted MRI at the level of the optic chiasm as an imaging parameter of the anterior perforated substance. The imaginary line (the Optic Chiasm – Insular Recess line) is drawn, from the optic chiasm medially to the insular recess laterally, to identify the anterior perforated substance (yellow line). The Porto Alegre line (Red Line) is as ascendent line from the lateral end of the OC-IR line and represents the lateral limit of the lateral LSTa inside the central core. In cases which the medial border of insular tumor crosses this line medially there is a great probability of involvement of the LSTa by the tumor.

**Figure 10:**
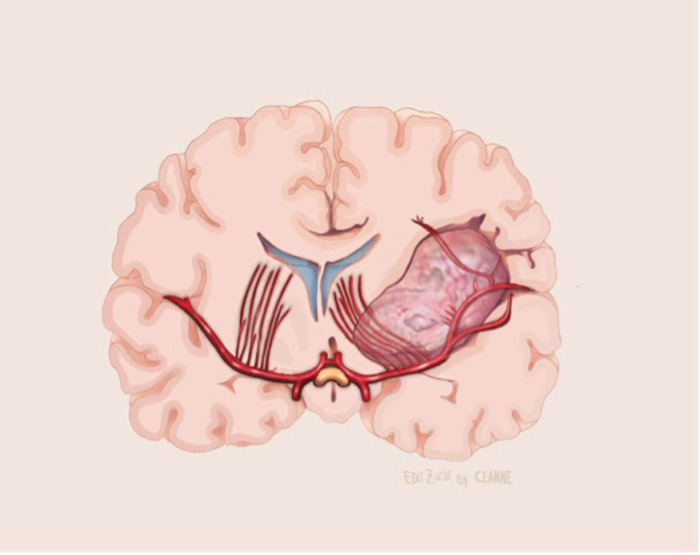
Illustrative drawing of coronal section showing the lenticulostriate arteries involvement by the medial portion of the insular glioma.

Regarding the lateral lenticulostriate arteries (LLAs) (Figures 8–10), these originate from the inferomedial region of the M1 segment and run through the central and lateral portion of the anterior region of the perforated substance. They vascularize the innominate substance, putamen, pale globe, caudate nucleus, internal capsule, radiated crown and the lateral portion of the anterior commissure. LLAs vary in number from 1 to 15, with no direct relationship to the length of the M1 segment and the present quantity of these arteries. The origin of these arteries can vary, and usually arise from the inferomedial region of the M1 segment. However, they can originate from temporal or frontal branches, or from the upper and lower M2 segment trunks. LLAs do not communicate with the subarachnoid space.

### M2 segment

The M2 segment originates in the limen insulae, where the main bifurcation of the middle cerebral artery occurs or, in some cases, trifurcation. Distally to the curve, the upper and lower trunks launch branches that run through the insula to the periinsular sulcus where the M3 segment begins. These branches are called “arterial trunks”, from which the cortical arteries originate.

The branches of the M2 segment can originate, in addition to the superior and inferior trunks, the intermediate trunk, the early branches, or even the accessory middle cerebral artery. The branches of the superior trunk vascularize the transverse, accessory insular gyri, short gyri of the insula, the insular apex, and the anterior limiting sulcus of the insula. The branches of the lower trunk irrigate the long posterior gyri of the insula, the lower limiting sulcus and the limen insulae.

The early branches originate close to the bifurcation or trifurcation and supply any part of the insula, with the exception of the central sulcus. They vascularize part of the inferior as well as the anterior limiting sulcus and limb of the insula. The accessory middle cerebral artery is the most common variation of the middle cerebral artery. It originates in the anterior cerebral artery and ends in the orbitofrontal region. It irrigates the accessory and transverse insular gyri and the anterior limiting sulcus.

There are 12 cortical arteries and they launch branches into the insula, except for the temporopolar artery. These are: orbitofrontal, prefrontal, precentral, central, anterior parietal, posterior parietal, angular, temporooccipital, posterior temporal, middle temporal, anterior temporal and temporopolar arteries. The cortical arteries of the M2 segment can originate from both the upper and lower trunk. It is from the upper trunk that most of the cortical arteries originate with, on average, five arteries from the upper trunk and varying from two to seven. From the lower trunk, two to eight arteries can exit. However, in patients with a trifurcation, the intermediate trunk can also give rise to the cortical arteries, ranging from one to two.

**Figure 11:**
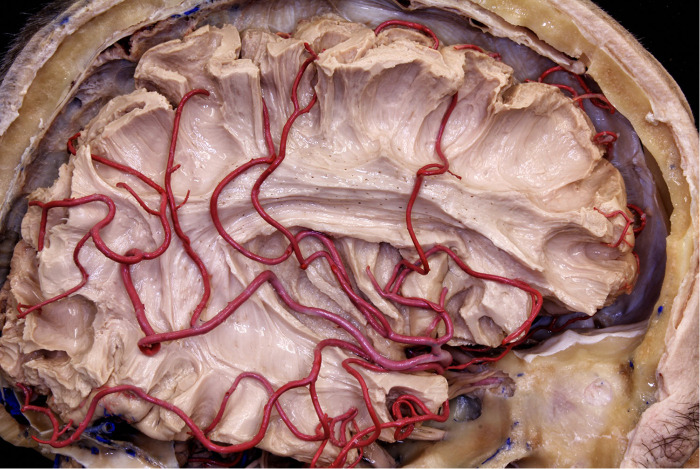
The MCA branches pass through the insular surface and irrigate most of the lateral surface of the brain. This is why all arterial branches passing through the insula must be preserved.

Cortical arteries that originate from the superior trunk generally travel a shorter path through the insula when compared to that of the inferior trunk arteries. In addition, they usually appear closer to the bifurcation, around the pole of the insula. The first cortical artery of the superior trunk is usually the orbitofrontal, followed by the prefrontal, precentral, central, anterior parietal, posterior parietal, angular and temporooccipital arteries. The anterior, central, temporooccipital and angular parietal arteries rarely originate from the inferior trunk. From the lower trunk, the cortical arteries pass through the long gyrus of the insula and the inferior limit of the sulcus and vascularize the posterior portion of the insula. Its first and most frequent branches are the middle and posterior temporal, followed by the anterior temporal, posterior parietal and temporopolar arteries (Figure 11).

**Figure 12:**
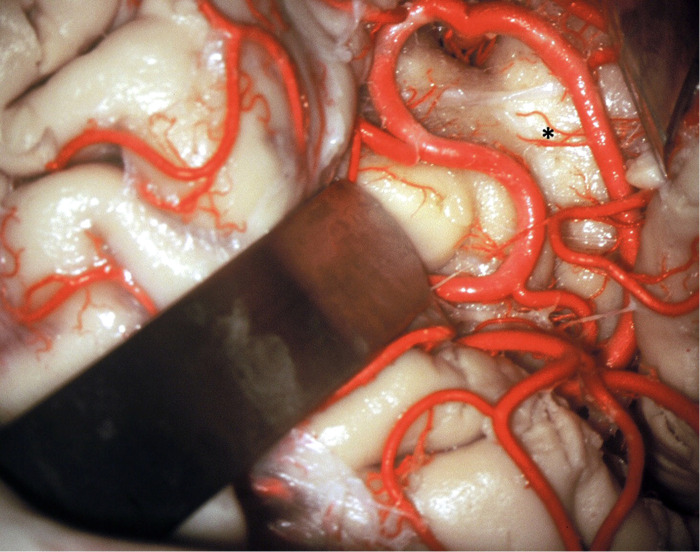
The vascularization of the insular cortex is provided by numerous small branches originating from the MCA M2 segment (*). In the resection of insular gliomas these branches can be coagulated.

Small branches of M2 penetrate the lateral surface to supply the insular cortex, the claustrum, and the external capsule. Approximately 80% to 90% of the insular arteries are short and vascularize the insular cortex and the extreme capsule (Figure 12). 10% are medium-length and are responsible for supplying the claustrum and external capsule; and the remaining 3% to 5% are long and reach the radiated corona. The latter are located mainly in the posterior region of the insula.

### M3 segment

The beginning of the M3 segment is marked by the anterior, superior, and inferior periinsular sulcus, and runs along the path to the medial surface of the operculum, that is, on the surface of the lateral sulcus where the M4 segment begins. Along its course, the M3 segment runs parallel to the M2, vascularizing the medial surface of the operculum. However, in some cases, the M3 segment can give rise to one or two small arteries, which are responsible for supplying the upper and lower periinsular sulci.

**Figure 13:**
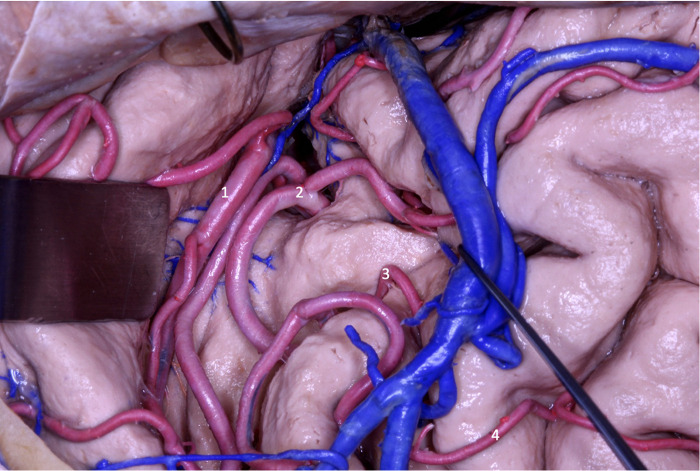
In the transsylvian approach, MCA segments M2 (1 and 2), M3 (3) and M4 (4) ACM are observed.

A peculiarity that the lateral orbitofrontal and temporopolar arteries may present is originating from the M1 segment and soon becoming the M3 segment while not extending any branches to the insula (Figure 13).

### M4 segment

The M3 segment runs sideways through the lateral sulcus, leaving it and becoming an M4 segment. This segment does not originate any branch for the insula and has no role in its vascularization.

### Overview of insular vascularization

For the vascularization the insular gyri, the pattern is as follows. The accessory and transverse gyri receive blood from the cortical arteries of the upper trunk. The orbitofrontal artery is the only one that vascularizes both gyri, except for the prefrontal artery which also vascularizes it to a lesser degree. The anterior short gyrus is supplied by the branches of the upper trunk and the prefrontal artery. The medium short gyrus is vascularized by the branches of the superior trunk and by the precentral artery, which is followed by the prefrontal artery. The apex of the insula receives blood from the cortical arteries of the superior trunk and from the prefrontal and precentral arteries. The posterior short gyrus receives branches from the superior trunk and central artery, which is accompanied by the precentral and anterior parietal arteries. The central sulcus and anterior long gyrus are the only regions of the insula that are vascularized by the branches of both the upper and lower trunk to the same degree, and are therefore combined in what is called the mixed vascularization zone. The central sulcus still receives blood from the central and anterior parietal arteries. The anterior long gyrus is also irrigated by the anterior and posterior parietal arteries. The posterior long gyrus is nourished by the branches of the lower trunk which are responsible for vascularizing this gyrus in 80% of the hemispheres. The angular and temporooccipital arteries supply this gyrus exclusively.

As for vascularizing the circular sulci of the insula, the pattern is as follows. The anterior circular sulcus receives blood from the branches of the upper trunk and from the orbitofrontal and prefrontal arteries. These exclusively vascularize this sulcus. The inferior circular sulcus receives branches from the lower trunks in 80% of the hemispheres and from the preceding branches in 50% of the hemispheres. It is also irrigated by the temporooccipital and posterior temporal arteries. Perforated arteries can be found in this sulcus, mainly in its posterior region. The limen region is predominantly vascularized by the initial portion of the inferior trunk in more than 80% of the hemispheres and receives contributions from the early branches in one third of the hemispheres. The middle temporal artery is responsible for vascularizing 30% of the hemispheres, extending more branches to the limen insulae than to the inferior trunk.

### Venous relationships

The venous system has numerous variations and asymmetries. However, there are some generalities. The superficial venous system drains the superficial cortical area of the lateral sulcus, while the deep venous system drains the insula. Between these two systems, several anastomoses are found.

The superficial venous system functions primarily through the superficial sylvian vein as it is the widest vessel draining the posterior branch of the lateral sulcus. The superficial sylvian vein usually originates as a single trunk. However, it can also originate as two trunks, which come together before draining into the venous sinus.

The superficial sylvian vein, in 85% of the cases, drains into the sphenoparietal sinus. In the remaining cases, it can drain into the cavernous sinus or the sphenopetrosal sinus.

The veins that drain the regions along the posterior branch of the lateral sulcus are named after the portion they drain. The vein coming from the frontal region is called fronto-sylvian, from the parietal, the parieto-sylvian vein, and from the temporal, the temporo-sylvian vein. The superficial sylvian vein generally receives six fronto-sylvian, four parieto-sylvian, and five temporo-sylvian veins.

The fronto-sylvian veins principally drain the inferior frontal gyrus, the portion adjacent to the middle frontal gyrus and the lower portion of the precentral gyrus. They also drain part of the anterior and posterior short gyri, the anterior long gyrus and the central, precentral and anterior circular sulci. In addition, in 80% of the hemispheres, they drain the middle short gyrus and the apex of the insula. Fronto-sylvian veins, in most hemispheres, drain into the superficial sylvian vein. However, in hemispheres where they do not drain into this vein, they empty into the Trolard vein that converges with the superior sagittal sinus. There are more anastomoses in the island veins than in the parieto-sylvian and temporo-sylvian veins.

The parieto-sylvian veins usually drain the postcentral gyrus and the inferior parietal lobe. In the two hemispheres, they also contribute to the drainage of the anterior long gyrus and the central insular sulcus. With roughly the same frequency, these either empty into the superficial sylvian vein or into the veins that enter the superior sagittal sinus.

The temporo-sylvian veins drain a larger region than the fronto-sylvian and parieto-sylvian veins. This region extends from the temporal pole to the final posterior part of the lateral sulcus. They also contribute to the venous drainage of the posterior long gyrus and the lower limit of the sulcus. In 15 hemispheres we examined, they drained into the superficial sylvian vein. As for the rest, they emptied into both the superficial sylvian vein and the vein of Labbe (Figures 4, 5).

The insula is drained mainly by the deep venous system, which is composed of the insular veins and the deep middle cerebral vein. However, in some areas, it can be drained by tributaries of the superficial sylvian vein.

The insular veins lead predominantly to the deep middle cerebral vein. However, they have anastomoses with the superficial sylvian vein in most of the hemispheres we studied.

According to Tanriover and collaborators (27), the drainage of the insula can be classified into three groups according to the venous system into which the area of the insula drains: superficial, deep and transitional. The transitional group drains into the deep venous system, and drains somewhat more into the superficial venous system.

The group that drains into the deep venous system corresponds to the limen area, inferior circular sulcus, long gyri and central sulcus. The superficial short venous system drains mainly the middle short gyrus and the apex of the insula. The transition zone includes the short anterior and posterior gyri and the anterior limit of the sulcus.

The insular veins are named according to the area they drain: anterior, precentral, central and posterior.

The deep middle cerebral vein, which is part of the deep venous system, is formed by the union of insular veins in the limen area. It is generally the anterior, precentral, central and posterior island veins that form part of the common transverse trunk.

## Discussion

### Critical reflexions of insular lobe surgical anatomy regarding intraoperative findings

#### Insula

Unlike vascular diseases related to the insula (cavernomas, MCA aneurysms), in which the anatomy of the sulci and gyri can be defined intraoperatively, in insular gliomas, this individualization of the anatomical structures is almost never possible, as the gyri are swollen by the tumor and lose their individuality. In the transsylvian approaches, the anatomical parameters for delimiting the insula in tumors are best provided by the superior and inferior circular sulci. Generally, the superior and inferior trunks of the middle cerebral artery lead to the superior and inferior circular sulci, respectively. In transcortical approaches, it is important to recognize that through the inferior frontal gyrus the surgeon will reach the upper part of the insula and through the superior temporal gyrus, the lower half of the insula (Figure 3). Is the transcortical approach, it is crucial to emphasize (as opposed to the transsylvian approach) the integrity of the arachnoid protecting the M2 branches of the MCA. This is especially useful to avoid pain (which can be excruciating) during the manipulation of these vessels in the transsylvian approach in awake surgery. The branches of the MCA are easily identified in the transcortical approach, but only at the end of the surgery, after the tumor is resected. In this way, the MCA is not manipulated during the procedure.

For gliomas located in the position II of Sanai and Berger (24–26), especially when the superior and posterior part of the tumor is prominent, cortical stimulation in the posterior portion of the inferior frontal gyrus is mandatory at a transcortical approach. Due to the neuroplasticity caused by low-grade gliomas, these areas of the inferior frontal gyrus (including Broca's area) can be resected, as they will almost always be silent.

For recurrent tumors, the surgeon must be careful not to coagulate the MCA M4 branches that are attached to the dura that is being opened, as these are generally *en passage* vessels directed to the cortex. This is especially important in the frontal lobe, in which the lesion of the M4 branches can cause ischemia and language impairment.

#### Lateral sulcus or sylvian fissure

According to Yasargil there are four types of sylvian fissures, some easier than others to dissect. There is no preoperative examination that predicts the degree of adhesion of the fontal and temporal lobes, and the surgeon must be prepared to deal with a more difficult fissure that will consume more time to be widely dissected. The average time of wide dissection of the sylvian fissure in an atraumatic and skillful way varies in around 1 h. The same is true for transcortical approaches. In order to avoid the sylvian fissure, the surgeon can perform the transcortical approach. There is no preoperative functional exam that precisely defines the site of the gyrus that can be entered. This is also a finding that will only be defined intraoperatively through cortical and subcortical stimulation.

#### M1 segment

In insular glioma surgery, the MCA M1 segment can be identified at the beginning (transsylvian approach) or at the end (transcortical approach) of surgery. One of the factors underdiscussed in the literature is the involvement of the lenticulostriate arteries by the medial part of the tumor. In our experience of 52 patients (article submitted to publishing), LSTa were founded to be involved by the tumor in 13 cases. In 39 patients, there were no involvement of the LSTa, which allowed a more aggressive resection.

We assume that early preoperative identification of the anterior perforated substance on the MRI and its proximity to the tumor may help determine the route of the LSTa over the medial tumor boundaries. The anterior perforated substance may be identified on coronal T2-weighted MRI on a slice through the optic chiasm. The optic chiasm is the medial reference, and the insular recess at the anteroinferior aspect of the insular pole is the lateral limit (Optic Chiasm - Insular Recess line – OC-IR line or Porto Alegre Line). We thus trace a vertical plane from the anterior perforated substance parallel to the median sagittal plane as the probable LSTa trajectory (Figures 9, 10).

**Figure 14:**
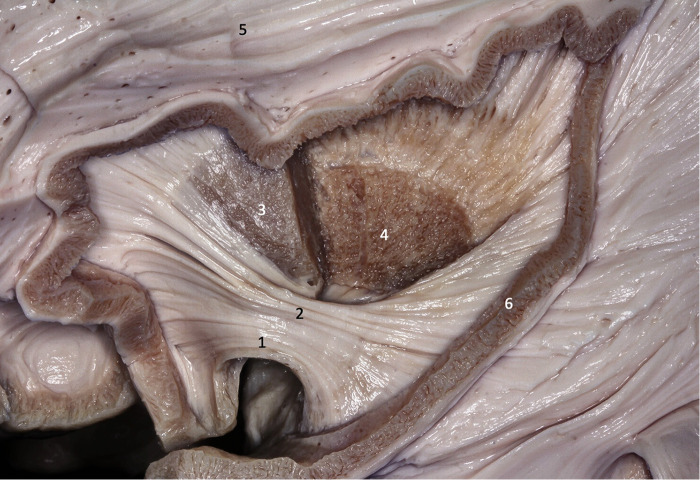
White fibers dissection of left cerebral hemisphere. The IFOF and the uncinate fasciculus form the lateral boundary of the beginning of the lenticulostriate arteries emergence (not shown in this dissection) near the limen of the insula. 1 - uncinate fasciculus; 2 - inferior fronto-occipital fasciculus; 3 - lentiform nucleus; 4 - globus pallidus; 5 - superior longitudinal fasciculus; 6 - inferior circular sulcus of insula.

**Figure 15:**
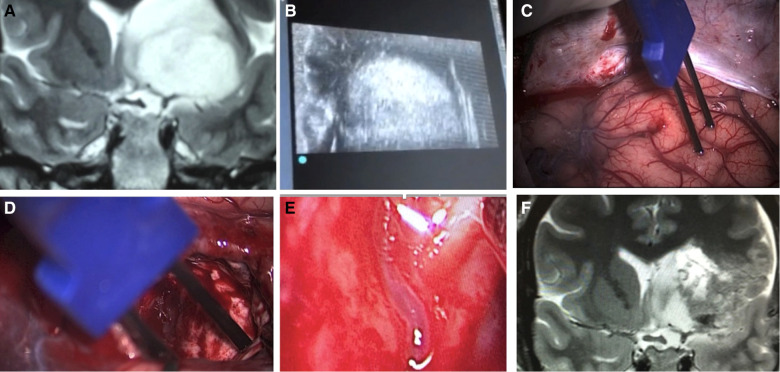
A case of insular glioma treated with LSTa as a resection limit. A - coronal T2-weighted MRI showing left-sided insular glioma with extension over the OC-IR line, suggesting a high probability of LSTa tumoral encasement; B - intraoperative echography as a tool to delineate the subcortical tumoral location; C - cortical brain mapping; D - subcortical brain mapping; E - intraoperative view of the LSTa within the tumor; F - postoperative MRI showing residual tumor beyond the OC-IR line, as it was the medial resection limit.

**Figure 16:**
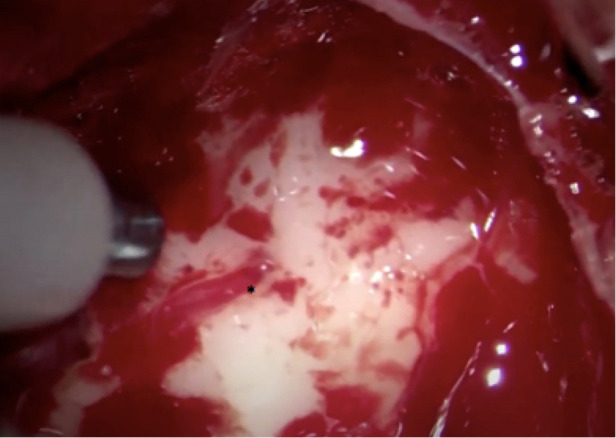
Intraoperative image showing LSTa surrounding by the tumor. The lenticulostriate artery (*) is the tumor medial resection limit.

**Figure 17:**
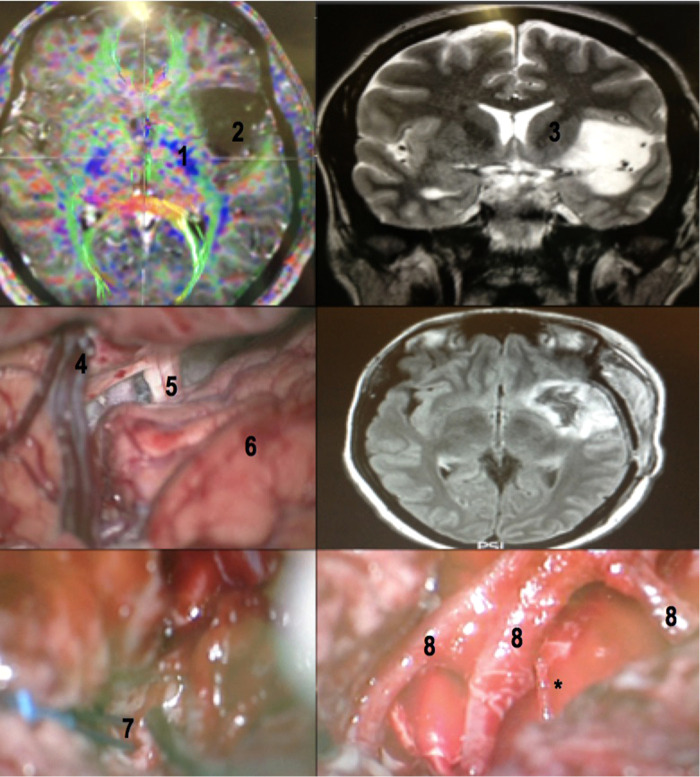
A 32-year-old female patient with drug-refractory epilepsy (10 to 15 complex partial seizures a day with no improvement with 02 monotherapy and 02 polytherapy anticonvulsants). Patient priorly submitted to a biopsy in another service, whose histopathology revealed grade II glioma. Awake surgery with cortical and subcortical mapping was performed with resection >90% of the tumor. Patient had a mild immediate postoperative speech deficit (paraphasia), which fully recovered with neuropsychological and speech therapy in 6 months. No postoperative motor deficits. No seizures (Engel 1) after surgery. MRI with tractography revealing the relation between the insular glioma and the internal capsule (upper left). Coronal T2-weighted MRI revealing the exact insular tumoral topography with a small temporal extension (upper right). The dissection of the sylvian fissure starts posteriorly and extends to the basal cisterns (central left). Flair MRI in the immediate postoperative period revealing tumor resection (central right). Subcortical stimulation to establish medial resection limit of tumor (lower left) and M2 branches after glioma resection (lower right). Note the large caliber perforating branch coming from the most posterior portion of one of the M2 branches (asterisk), which in these cases must be preserved. 1- internal capsule (viewed in a tractography (MRI)); 2 - insular glioma (MRI); 3 - internal capsule; 4 - superficial sylvian vein draining to the sphenoparietal venous sinus; 5 - optic nerve; 6 - frontal lobe; 7 - bipolar stimulator; 8 - MCA M2 branches.

For patients of insular gliomas undergoing awake surgery, our medial limit of resection (namely the tumor part located next to the limen insula) was identified primarily through altered speech patterns, such as paraphasia, during electric subcortical stimulation of the inferior fronto-occipital fasciculus (IFOF) (Figure 14). To evaluate the potential involvement of LSTa within the medial aspect of the tumor, we carried out an accurate analysis of coronal and axial T2 and T1-weighted MR images to consider that the tumors that entangled these vessels could not be entirely resected. In cases in which there was a likelihood of involvement of the LSTa, the parameter used to stop the resection of the medial component of the tumor was the direct intraoperative visualization of the LSTa. At the time of visualization of the LSTa within the tumor, resection was interrupted. In cases where it was assumed that the LSTa were not involved, the parameter used to stop the medial part of resection was the confirmation of the corticospinal tract with a 10-mA stimulus (Figures 15–17).

#### M2 segment

For a surgical purpose, it is not necessary identify the 12 cortical MCA branches. On the other hand, much has been said about the long perforating branches of the M2 segment to the insula cortex. From an anatomical point of view, there are long perforating branches (Long Perforating arteries (LPra), commonly located on the posterior half of the central insular sulcus and the long gyri, that can reach the corona radiata and/or deeper white fibers in up to 36% of cases (29). Although this is an interesting anatomical finding, in our cases of transsylvian approach we have always coagulated the branches that perforate the insular cortex both at the level of the short gyri (anteriorly) and at the level of the long gyri (posteriorly). This maneuver was always performed, without any motor sequelae in our patients (Figure 17).

When dealing with insular tumors, there is no need to accurately identify this entire surgical anatomy. What the surgeon needs to keep in mind is that all arterial branches (except the small branches that supply the insula and originate from M2) must be preserved. While in the transsylvian approach (which we use for type IIIA and IIIB tumors of the Yasargil's classification) these branches are identified early and preserved, in the transcortical approach these branches are visualized many times at the end of the tumor resection. For this reason, it is very important to leave the ultrasonic aspirator set with low aspiration and low power, so that there is no unintentional injury to these arteries.

#### Venous relationships

Generally, the superficial (sulci and gyri) and deep insular veins can be freely coagulated. The great challenge that arises in some cases is the opening of the posterior third of the sylvian fissure. In some cases, we have observed a rich venous anastomotic complex (sometimes including Trolard and Labbé veins) that prevents extensive dissection of the posterior third of the sylvian fissure, as it would be possible to injure these important veins. In some cases, we have to decide, at that moment, to switch the transsylvian to a transcortical approach, awaking the patient for cortical and subcortical mapping, to prevent speech impairment if it is in the dominant hemisphere. It is believed that the coagulation of these veins can lead to venous infarction in 30% of patients.

### Arterial relationships

Some key factors influence the extent of resection on the medial face of insular tumors. The insular cortex faces laterally and forms the medial wall of the operculoinsular compartment. The gray matter structures that lie medial to the insular cortex include claustrum and the putamen; more medial is the internal capsule, easily visible on MRI of healthy subjects. When tumor is present, the claustrum is challenging to see because of the slim shape and weak structure. The putamen has a firmer structure than the insular cortex and can usually resist glioma spreading. On MRI images, although some insular tumors show clear limits, other seems to invade the putamen, and even the internal capsule. The branches of the MCA (M2) usually can be identified laterally to the insular surface. Small branches of M2 penetrate the lateral surface to supply the insular cortex, the claustrum, and the external capsule. The LSTa (lateral group) are branches of the M1 segment (sphenoidal) and cross superiorly to penetrate the anterior perforated substance to supply blood to the substantia innominata, putamen, globus pallidus, head and body of caudate nucleus, internal capsule and adjacent corona radiata, and the lateral portion of the anterior commissure. They are numbered one to 15 (average 7.75) per hemisphere. The diameter of LSTa range from 0.1 to 1.5 mm. Usually, there is no anastomosis between any LSTa. Lateral view of the LSTa reveals a fanlike appearance over the lateral surface of the internal capsule (27).

The sylvian fissure must be opened, or a trans-opercular route must be carried out, to expose the insular glioma. As tumor removal progresses medially, the LSTa can be compromised, leading to ischemia of the basal ganglia and internal capsule (19, 20). So, it is of paramount importance to preserve the LSTa. Some tumors may grow and infiltrate medial structures including the LSTa. In this case, it is recommended to consider stopping the resection, reckoning the LSTa as a primary limiting factor. For a safe insular tumor resection, the medial limit of resection is defined by the vertical plane formed by the intraparenchymal course of the LSTa

According to Türe and Yasargil (21), the M1 segment, in 38 of the 40 hemispheres, gave rise to one to three cortical arteries, which were located, in most cases (75.8%), laterally to this segment and suppled the temporal lobe. However, cortical arteries also originated from the medial region (24.2%), vascularizing the frontal lobe. In seven hemispheres, small cortical arteries were found irrigating the piriform cortex (30).

Cortical branch variations can be classified into types A, B, C, and D, according to the region of the M1 segment from which they originate. In type A, this segment only separates into temporal (lateral) cortical branches and may vary in number from one to three, the most common being just one branch. In type B, the M1 segment gives rise to both temporal and frontal (medial) branches. In this type, the most encountered variation is the origin of a temporal branch and a frontal branch, though two temporal branches and a frontal branch can also be found. In type C, the M1 segment gives rise to frontal branches only. In type D, the cortical branches do not originate from this segment, only the lateral lenticulostriate arteries and arteries of the uncus. Of all these types, type A is the most common, and types C and D the rarest.

In Moshel series (13), the location of the displaced LSTa on preoperative angiograms was used to identify tumors that extend medially into the putamen and globus pallidus and present a more significant risk for postoperative­ deficit.­ They­ found­ out that­ this­ extension­ was highly correlated ­when­ the­ MRI defined­ medial ­tumor boundaries superimposed onto stereotactic cerebral arteriograms. In their series of 38 insular gliomas patients, a gross total resection was achieved more frequently when tumors were lateral to the LSAs, shifted the LSAs medially, and had a well-defined tumoral border on­ T2-weighted­ MR­ images.­ In cases where tumors extended medial to the LSTa the EOR was subtotal.

Intraoperative identification of the LSTa in the sylvian vallecula does not reflect necessarily their intraparenchymal course because the tumor may displace such arteries medially (13, 17, 18). Yaşargil observed that low-grade gliomas initially spread within the anatomic limbic system's confines, suggesting that tumor resection is complete when LSTa are seen intra-operatively or when the ordinary white matter overlying the putamen is encountered (4).

In our reflections, we introduced our imaging and anatomical concept regarding LSTa in insular glioma surgery. Accurate identification of origin, route, and distribution of the LSTa is pivotal to surgical success, especially the lateral group (32, 33). Their identification or, at least, the anatomical knowledge of their path impacts directly on the extent of tumor resection and on functional preservation. After originating from the dorsal aspect of the MCA M1 segment, the LSTa penetrate the lateral two-thirds of the anterior perforated substance in the basal forebrain, a quadrilateral area of grey matter positioned posterior to the gyrus rectus and olfactory trigone and anterolateral to the optic tract. It is bounded medially by the optic chiasm and laterally by the lateral olfactory striae. After crossing the anterior perforated substance, the LSTa distribute to lateral thirds of the anterior commissure, lateral part of the globus pallidus, superolateral two-thirds of the head and entire body of the caudate nucleus and most of the putamen out to and including the external capsule, superior part of the entire anterior limb and superior part of the posterior limb of the internal capsule and periventricular white matter (corona radiata) at the angle of the lateral ventricle.

Isolated tumor cells in insular gliomas can infiltrate intact parenchyma and form tumoral tissue that encases the LSTa (17, 18, 34–37). Some authors have advocate stereoscopic angiography and computerized tumor reconstruction (31), MR angiography, and CT angiography to predict the proper position of the LSTa. Despite the elegance of these studies, we believe that MRI T2-weighted imaging (OC-IR line) can anticipate the LSTa's position and course for practical purposes. This method could find LSTa anatomically and medially encased by the tumor in 13 subjects (25%). In 75% of the patients the tumor medial border was restricted to the normal white matter lateral to the putamen. In such latter cases, the tumor was resected over 90%. LSTa involvement was a limiting factor to a reasonable extent of resection and we advocate that tumors encasing such arteries must be left behind because of high risk of permanent postoperative functional deficit.

Another possible etiology of internal capsule and corona radiata infarction could be, at least in theory, coagulation or lesion of the Long Perforating arteries (LPra) (29, 32, 33). The LPra are those arteries originating from the M2 segment of the MCA that vascularize the insular cortex. Most of these arteries are short. However, these LPra, most commonly located on the posterior half of the central insular sulcus and the long gyri, can reach the corona radiata and/or more profound the fibers in 36% of the cases. Some of these crossed the fibers of the corona radiata and joined the lateral ventricular body's ependyma. The external capsule is the border of the territories supplied by the short insular arteries. There is no anastomosis between the insular arteries and the lenticulostriate arteries. Although the LPra could explain a postoperative motor deficit after insular glioma surgery with LSTa preservation, we did not observe it in any of our patients, even coagulating all insular perforator arteries during the transsylvian approach (Figure 17).

## Conclusion

When medical science deals with rare diseases, such as insular gliomas, the highest level of evidence we can have is 3, the result of case series. Due to the fact that knowledge of microsurgical anatomy, brain mapping and surgical experience count a lot in this type of surgery, it is difficult to create a rational procedure flowchart to be taken intraoperatively. For this reason, the reflections discussed here are the basis of the microsurgical anatomy of the possible vascular complications in insular glioma surgery.

## Data Availability

The raw data supporting the conclusions of this article will be made available by the authors, without undue reservation.
